# Supplementary material for: Exceptional non-Hermitian topological edge mode and its application to active matter
Source: Nat Commun. 2020 Nov 12;11:5745. doi: 10.1038/s41467-020-19488-0 (PMC7665040; doi:10.1038/s41467-020-19488-0)
Supplement: Supplementary file 1 — Supplementary Information [file 41467_2020_19488_MOESM1_ESM.pdf]

Supplementary Information

Exceptional non-Hermitian topological edge mode and its application to active matter

Sone et al.

Supplementary Methods - Tight-binding models in real-space basis

Here we explicitly describe the Hamiltonian used in the main text. The Hamiltonian of the Qi-Wu-Zhang (QWZ) model [1] in the real-space basis is denoted as

$$\begin{aligned} H_{\mathrm{QWZ}} = & \sum_x \sum_y \left(|x+1, y\rangle\langle x, y| \otimes \frac{\sigma_z + i\sigma_x}{2} + \mathrm{h.c.} \right) \\ & + \sum_x \sum_y \left(|x, y+1\rangle\langle x, y| \otimes \frac{\sigma_z + i\sigma_y}{2} + \mathrm{h.c.} \right) + u \sum_x \sum_y |x, y\rangle\langle x, y| \otimes \sigma_z, \end{aligned} \quad (1)$$

where h.c. represents the Hermitian conjugate of the previous term and σ_i is the ith component of the Pauli matrices. This model contains two sublattices. To construct a minimal model for exceptional edge modes, we prepare two layers of the QWZ model to construct a higher Chern number insulator as is done in the previous studies [2, 3]. Then, we combine them with their time-reversal counterparts by non-Hermitian and Hermitian couplings. The obtained Hamiltonian can be written by a 4×4 matrix form as

$$H = \begin{pmatrix} H_{\mathrm{QWZ}} & cI_2 & ia\beta\sigma_x & -i\gamma\sigma_x \\ cI_2 & H_{\mathrm{QWZ}} & i\gamma\sigma_x & ia\beta'\sigma_x \\ ia\beta\sigma_x & -i\gamma\sigma_x & H^*_{\mathrm{QWZ}} & cI_2 \\ i\gamma\sigma_x & ia\beta'\sigma_x & cI_2 & H^*_{\mathrm{QWZ}} \end{pmatrix}. \quad (2)$$

In the numerical calculations of Fig. 1b and 3 in the main text, we use the parameters $u = -1$, $c = 0.2$, $\beta = 0.14$, $\beta' = 0.06$, $\gamma = 0.05$, and $a = 1$. Also in the numerical calculation of Fig. 2 in the main text, we use the parameters $u = -1$, $c = 0.2$, $\beta = 0.9$, $\beta' = 0.7$, and $\gamma = 0.8$ and change the parameter a.

Supplementary Note 1 - Absence of imaginary parts of eigenenergies in the two-layered Bernevig-Hughes-Zhang model

We discuss why the bulk bands of the two-layered Bernevig-Hughes-Zhang model exhibit only the real eigenenergies (cf. Eq. (1) and Fig. 1b in the main text). We can directly show that the imaginary parts of the bulk eigenenergies become zero at least in the range of the first-order perturbation with respect to the non-Hermitian coupling. If we describe the QWZ Hamiltonian as $H = \mathbf{R} \cdot \boldsymbol{\sigma}$, where $\mathbf{R} = (\sin k_x, \sin k_y, u + \cos k_x + \cos k_y)^T = |R|(\sin\theta\cos\phi, \sin\theta\sin\phi, \cos\theta)^T$ and $\boldsymbol{\sigma} = (\sigma_x, \sigma_y, \sigma_z)^T$, we obtain the explicit forms of the

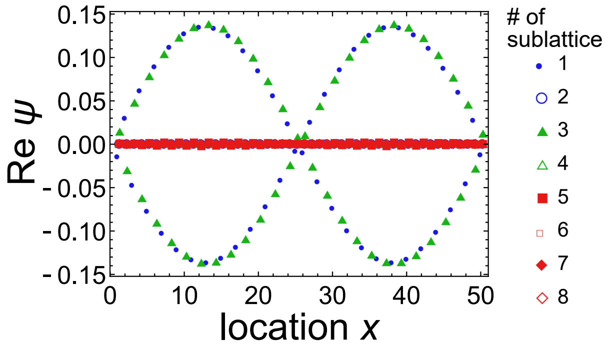

Supplementary Figure 1. **Bulk eigenmode in the two-layered non-Hermitian Bernevig-Hughes-Zhang model.** The bulk eigenmode is calculated under the open boundary condition in the x direction and the periodic boundary condition in the y direction. We align 50 sites in the x direction. The legend shows the correspondence between each mark and the sublattice (eight per site). The parameters used are the same as in Fig. 1 in the main text.

eigenvectors of the QWZ model,

$$|+, \mathbf{R}(\mathbf{k})\rangle = e^{-\frac{i\psi}{2}} \begin{pmatrix} e^{-\frac{i\phi}{2}} \cos\frac{\theta}{2} \\ e^{\frac{i\phi}{2}} \sin\frac{\theta}{2} \end{pmatrix}, \tag{3}$$

$$|-, \mathbf{R}(\mathbf{k})\rangle = e^{-\frac{i\psi}{2}} \begin{pmatrix} e^{-\frac{i\phi}{2}} \sin\frac{\theta}{2} \\ -e^{\frac{i\phi}{2}} \cos\frac{\theta}{2} \end{pmatrix}. \tag{4}$$

By utilizing these expressions, we can obtain the eigenvectors of the two-layered QWZ model,

$$|+, \pm, \mathbf{R}(\mathbf{k})\rangle = \frac{1}{\sqrt{2}} e^{-\frac{i\psi}{2}} \begin{pmatrix} e^{-\frac{i\phi}{2}} \cos\frac{\theta}{2} \\ e^{\frac{i\phi}{2}} \sin\frac{\theta}{2} \\ \mp e^{-\frac{i\phi}{2}} \cos\frac{\theta}{2} \\ \mp e^{\frac{i\phi}{2}} \sin\frac{\theta}{2} \end{pmatrix}, \tag{5}$$

$$|-, \pm, \mathbf{R}(\mathbf{k})\rangle = \frac{1}{\sqrt{2}} e^{-\frac{i\psi}{2}} \begin{pmatrix} e^{-\frac{i\phi}{2}} \sin\frac{\theta}{2} \\ -e^{\frac{i\phi}{2}} \cos\frac{\theta}{2} \\ \mp e^{-\frac{i\phi}{2}} \sin\frac{\theta}{2} \\ \pm e^{\frac{i\phi}{2}} \cos\frac{\theta}{2} \end{pmatrix}. \tag{6}$$

On the other hand, we note $H^*_{\mathrm{QWZ}}(-\mathbf{k}) = \mathbf{R}'(\mathbf{k}) \cdot \boldsymbol{\sigma}$ with $\mathbf{R}'(\mathbf{k}) = (-\sin k_x, \sin k_y, u + \cos k_x + \cos k_y)$. If we rewrite $\mathbf{R}'$ as $|R'|(\sin\theta' \cos\phi', \sin\theta' \sin\phi', \cos\theta')$, we obtain $\theta'(\mathbf{k}) = \theta(\mathbf{k})$, $\phi'(\mathbf{k}) = \pi - \phi(\mathbf{k})$. The first-order perturbation by non-Hermitian and Hermitian

couplings can be obtained from a matrix whose elements are $\langle \pm, \mathbf{R}(\mathbf{k})|\sigma_x|\pm, \mathbf{R}'(\mathbf{k})\rangle$, and we can calculate them as, for example,

$$\begin{aligned}\langle +, \mathbf{R}(\mathbf{k})|\sigma_x|+, \mathbf{R}'(\mathbf{k})\rangle &= e^{i\frac{\phi+\phi'}{2}} \cos\frac{\theta}{2}\sin\frac{\theta'}{2} + e^{-i\frac{\phi+\phi'}{2}} \sin\frac{\theta}{2}\cos\frac{\theta'}{2} \\ &= (e^{i\frac{\pi}{2}} + e^{-i\frac{\pi}{2}})\cos\frac{\theta}{2}\sin\frac{\theta}{2} = 0. \end{aligned} \tag{7}$$

Therefore, the first-order perturbation becomes zero for the bulk bands and thus does not generate nonzero imaginary parts of the eigenenergies.

We can justify the use of the Bloch Hamiltonian for the purpose of predicting the bulk eigenstates under the open boundary condition, while recent studies [4–8] have revealed that some non-Hermitian systems breaking the bulk-edge correspondence exhibit bulk modes different from ones predicted from their Bloch Hamiltonians. We numerically calculate the bulk eigenstates and confirm the consistency with the prediction from the Bloch Hamiltonian. Supplementary Figure 1 shows an example of bulk eigenstates. It resembles the Bloch wave (i.e. sine curve). Besides, its probabilistic density is localized at the half of sublattices, which implies that two bulk eigenmodes obtained from the QWZ model and its time-reversal counterpart are separated under the small non-Hermitian coupling. These behaviors of the bulk eigenstates are consistent with the discussion in the previous paragraph. Therefore, we can expect that the Bloch Hamiltonian predicts the behavior of the bulk eigenmodes in the model under the open-boundary condition. We note that the breakdown of the bulk-edge correspondence discussed here indicates the existence of the unpredicted robust edge modes with the exceptional points (EPs) and does not imply the other context of the breakdown such as non-Hermitian skin effect [6–8]. In the previous paragraph, furthermore, we use the bulk eigenstates predicted from the Bloch Hamiltonian as the eigenstates of the nonperturbed Hermitian Hamiltonian and thus can expect that the perturbation calculation based on such nonperturbed eigenstates reach the proper conclusion.

We can also partially explain the reality of the eigenenergies of the bulk modes from the PT symmetry of the system. The PT symmetry of the Hamiltonian $H(\mathbf{k})$ of the two-layered non-Hermitian Bernevig-Hughes-Zhang model is described as

$$PT = \sigma_x \otimes I_2 \otimes \sigma_z, \quad PTH(\mathbf{k})(PT)^{-1} = H^*(\mathbf{k}). \tag{8}$$

The PT symmetry guarantees that the eigenvalues become real or appear as pairs of complex conjugates. However, it is impossible to predict which of those behaviors appears.

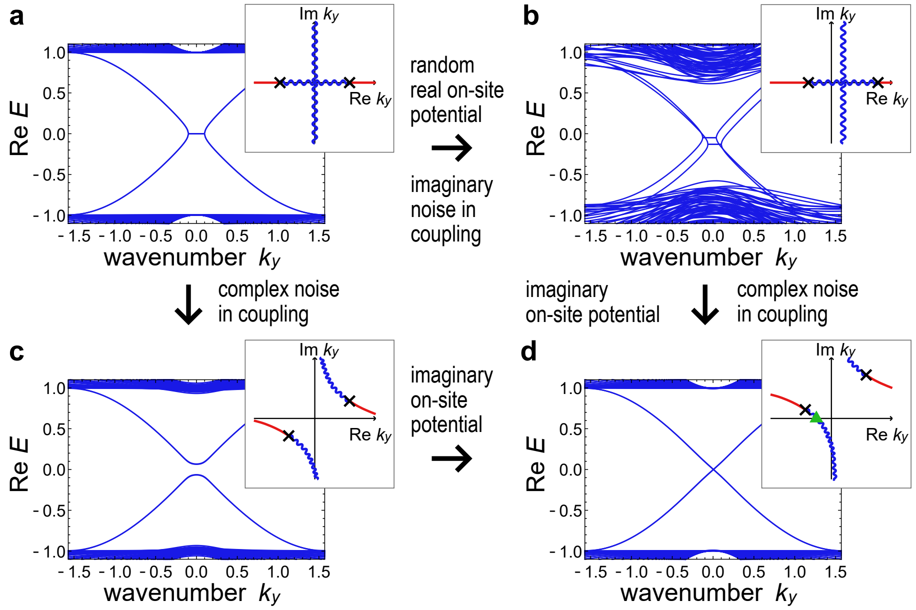

Supplementary Figure 2. **Edge modes in disordered nontrivial systems and their robustness.** **a-d,** Each inset shows the exceptional points (EPs) (black crosses) and the curve of the degeneracy of the real (blue wave curves) and imaginary (red curves) parts of the edge eigenenergies in the complex wavenumber plane as in Fig. 3 in the main text. **a,** The main panel shows the obtained exceptional edge modes in the nontrivial system (Supplementary Eq. (9)) without disorder. The parameters used are $u = -1$ and $\beta = 0.1$. **b,** The main panel shows the edge dispersion with on-site random real potentials and imaginary disorder in the coupling term. There still exist EPs and edge modes in the bulk energy gap as in the model in the main text. The noise width is set to be $W = 0.5$ ($W = 0.02$) for the random real on-site potential (the imaginary noise in the non-Hermitian coupling). **c,** The gap is opened and the edge modes no longer exist in the system with random Hermitian couplings. The noise width is set to be $W = 0.1$ for the real noise in the non-Hermitian coupling. **d,** When we add imaginary on-site potentials, the edge modes are recovered even under the random Hermitian couplings, while EPs disappear from the edge modes. The noise width for the real noise in the non-Hermitian coupling is the same as in panel **c** and the strength of the on-site imaginary potential is $g = 0.2$.

Supplementary Note 2 - Exceptional edge modes in a nontrivial tight-binding model

Here, we discuss the existence and the robustness of exceptional edge modes in topologically

nontrivial systems, while we concentrate on the systems with topologically trivial bulks in the main text. To construct the topologically nontrivial model, we again utilize the construction procedure for time-reversal-symmetric topological insulators. We combine the QWZ model and its time-reversal counterpart by a non-Hermitian coupling and obtain the Hamiltonian,

$$\begin{aligned} H &= \begin{pmatrix} H_{\mathrm{QWZ}} & i\beta\sigma_x \\ i\beta\sigma_x & H^*_{\mathrm{QWZ}} \end{pmatrix} \\ &= (u + \cos k_x + \cos k_y) I_2 \otimes \sigma_z + \sin k_y I_2 \otimes \sigma_y + \sin k_x \sigma_z \otimes \sigma_x + i\beta\sigma_x \otimes \sigma_x \end{aligned} \tag{9}$$

where β is real. This Hamiltonian exhibits the time-reversal symmetry and the pseudo-Hermiticity, and we can obtain both the nontrivial $\mathbb{Z}_2$ and $\mathbb{Z}$ invariant defined in the time-reversal symmetric and the pseudo-Hermitian systems. ($\mathbb{Z}$ invariant can be calculated from the Chern number of ηH [9, 10], where η is the operator determining the pseudo-Hermiticity of the Hamiltonian H.) Therefore, the edge modes appear in this model are protected by the topology and the symmetries of the system. However, the EPs in the edge bands protect the edge modes from the gap openings against disorders breaking the time-reversal symmetry and the pseudo-Hermiticity. To confirm such protection, we numerically calculate the edge band structures under the existence of disorders. As in Fig. 3 in the main text, we consider a random real on-site potential, imaginary and real noises in the non-Hermitian coupling and an imaginary on-site potential expressed as follows:

$$a(x)\{I_2, \sigma_z\} \otimes \{I_2, \sigma_z\} \quad \text{(random real on-site potential)} \tag{10}$$

$$b(x)\{\sigma_x, i\sigma_y\} \otimes \{\sigma_x, i\sigma_y\} \quad \text{(noise in the non-Hermitian coupling)} \tag{11}$$

$$ig\sigma_z \otimes I_2 \quad \text{(imaginary on-site potential)} \tag{12}$$

where brackets mean that we introduce all the combinations made by choosing either one in each bracket. $a(x)$ and $b(x)$ are random values for each x from uniform distributions ranging $[-W, W] \in \mathbb{R}$ or $i[-W, W] \in i\mathbb{R}$. We set $a(x)$ to be real and $b(x)$ to be imaginary (real) for the imaginary (real) noise in the non-Hermitian coupling. Supplementary Figure 2 shows the results of the numerical calculations. We can confirm that all the results are consistent with the prediction from the effective edge Hamiltonian and the results of the numerical calculations on the disordered two-layered non-Hermitian Bernevig-Hughes-Zhang model (cf. Eq. (3) and Fig. 3 in the main text). We note that the random real on-site potential and the imaginary noise in the non-Hermitian coupling breaks both the time-reversal symmetry and

the pseudo-Hermiticity, while the exceptional edge modes exist robustly against these disorders. Therefore, exceptional edge modes are protected against symmetry-breaking disorder and thus improve the robustness of edge modes even in topologically nontrivial systems.

Supplementary Note 3 - Topological index for EPs in the complex wavenumber space

It has been proposed [11] that the topological invariants for EPs can be defined in the two-dimensional wavenumber space. In our models, however, EPs appear in the edge band structure for one-dimensional wavenumber k_y. Considering the complex wavenumber, we assume that the EPs appear in the two-dimensional parameter space of the real and imaginary parts of the wavenumber. Then, we can define the topological invariant for the EPs in the edge band structure as

$$\nu_{\pm} = \frac{1}{2\pi} \oint_{|k_y \mp \beta| = \delta} \frac{d}{dk_y} \arg[E^+(k_y) - E^-(k_y)] dk_y, \tag{13}$$

where $E^{\pm}(k_y)$ is the eigenenergy of the two bands around the EPs and δ is the small radius of the circular integral path. We note that in our model two edge eigenstates coalesce at each EP, while in general, three or more edge eigenstates can coalesce at one EP. In the latter case, we need to generalize the definition of the topological index to include the effect of all the relevant eigenvalues.

We next calculate the topological index of the EPs in the exceptional edge modes via the effective edge Hamiltonian $H(k_y) = E_0 I + k_y \sigma_z + i\beta' \sigma_x$, which describes exceptional edge modes in the disorder-free system. The Hamiltonian possesses the eigenenergies $E^{\pm}(k_y) = E_0 \pm \sqrt{k_y^2 - \beta'^2} = E_0 \pm \sqrt{k_y - \beta'}\sqrt{k_y + \beta'}$ and EPs at $k_y = \pm\beta'$. We can calculate the topological index around the EP at $k_y = \beta'$ as

$$\begin{aligned}\nu &= \frac{1}{2\pi} \oint \frac{d}{dk_y} \arg[2\sqrt{k_y - \beta'}\sqrt{k_y + \beta'}] dk_y \\ &= \frac{1}{2\pi} \int_0^{2\pi} \frac{d}{d\theta} \arg[2\sqrt{\delta e^{i\theta}}\sqrt{2\beta' + \delta e^{i\theta}}] d\theta \\ &= \frac{1}{2\pi} \int_0^{2\pi} \frac{d}{d\theta} [\theta/2 + \arg(2\beta' + \delta e^{i\theta})/2] d\theta \\ &= \frac{1}{2},\end{aligned} \tag{14}$$

where we consider a circle with a small radius δ centered at $k_y = \beta'$ as the integral path. Similarly, we can check that the topological index of the EP at $k_y = -\beta'$ is 1/2. These

topological indices are invariant under the continuum deformation of the Hamiltonian, thus indicating the robustness of the EPs in the complex wavenumber space.

Supplementary Note 4 - Perturbation analysis on the edge modes avoiding in the imaginary part of the energy

Here, we discuss the origin of the robustness of the edge modes in Fig. 3d in the main text, which apparently cross each other but have the different imaginary parts of the eigenenergies. We utilize the perturbation theory and the continuity of the band structure. Since each edge band is continuous with respect to the wavenumber k_y and the change of the parameter, edge bands connecting positive and negative bulk bands should remain gapless until two or more edge bands become degenerate. From the perturbation calculation, however, we can understand that such degeneracy is prohibited under a small disorder. Therefore, the gapless edge bands in Fig. 3d in the main text are robust against disorder.

To be concrete, we consider two edge modes, $|\psi_1(k_y)\rangle$ and $|\psi_2(k_y)\rangle$ with the eigenvalues $E_1(k_y)$ and $E_2(k_y)$, for each. We assume that the other eigenvalues are separated far from $E_1(k_y)$ and $E_2(k_y)$ and thus the other eigenvectors only have a negligible effect on the perturbed edge modes. The perturbation theory predicts that if the expectation value of the perturbation ϵV is much smaller than $|E_1(k_y)-E_2(k_y)|$, the eigenvalues of $H(k_y)+\epsilon V(k_y)$ corresponding to $E_1(k_y)$ and $E_2(k_y)$ are $E_1'(k_y)=E_1(k_y)+\mathcal{O}(\epsilon)$ and $E_2'(k_y)=E_2(k_y)+\mathcal{O}(\epsilon)$. Then the change of the distance of the two edge eigenvalues is $\mathcal{O}(\epsilon)$. Since this change is much smaller than the original distance $|E_1(k_y)-E_2(k_y)|$, two edge modes are always nondegenerate in adding perturbations. Therefore, reconfiguration of the two edge modes are prohibited, and the edge modes remain connecting the upper and lower bulk bands, which implies the existence of gapless edge modes in the real part of the eigenenergy.

Supplementary Note 5 - Spreading of the edge wave packet induced by the complex frequencies

In the real-space simulation of the topological insulator laser (cf. Fig. 5 in the main text and Supplementary movies 1-3), the edge wave packet gradually spreads. To understand the origin of such spreading, we consider the one-dimensional wave packet described as

$$u(x,t)=\int dk\phi(k)e^{i(kx-\omega(k)t)}. \tag{15}$$

By expanding the index around the wavenumber k_0, which maximizes $\mathrm{Im}\,\omega$, we obtain the following expression,

$$u(x,t) \simeq e^{i(k_0 x - \omega(k_0)t)} \int dk \phi(k) e^{i(x - v_g t)(k-k_0)} e^{\frac{\partial^2 \mathrm{Im}\,\omega}{\partial k^2}(k-k_0)^2 t}, \tag{16}$$

where $v_g = \frac{\partial \mathrm{Re}\,\omega}{\partial k}$ is the group velocity of the wave packet. The last exponential represents the spreading wave packet, which does not appear in the real dispersion. To see the spreading clearly, we consider $\phi(k) = 1$ and obtain the wave packet

$$u(x,t) = \sqrt{\frac{\pi}{At}} e^{i(k_0 x - \omega(k_0)t)} e^{-\frac{(x-v_g t)^2}{4At}}, \tag{17}$$

where $A = -\partial^2 \mathrm{Im}\,\omega / \partial \mathrm{k}^2 > 0$. This implies that the width of the wave packet increases in proportional to the square root of time.

We also calculate the group velocity and the spreading speed of the lasing wave packets described by the following edge effective Hamiltonian,

$$H_{\mathrm{edge}}(k) = \begin{pmatrix} E_0 + ak & i\beta \\ i\beta' & E_0 - k \end{pmatrix}, \quad a \neq 1. \tag{18}$$

This Hamiltonian exhibits the eigenenergies $E = E_0 + [(a-1)k \pm \sqrt{(a+1)^2 k^2 - 4\beta\beta'}]/2$ and the imaginary parts take the maximum or minimum values at the wavenumber $k = 0$. Therefore, the group velocity v_g and the spreading speed A are $v_g = (a-1)/2$ and $A = (a+1)^2/(4\sqrt{\beta\beta'})$.

Supplementary Note 6 - Two-layered Bernevig-Hughes-Zhang topological insulator laser

The two-layered Bernevig-Hughes-Zhang model (cf. Eq. (1) and Fig. 1b in the main text) exhibits the nonzero imaginary part of the eigenenergies only in the exceptional edge modes as is the case for the topological insulator laser [12, 13]. Thus, we can construct another topological insulator laser by modifying that model. The Hamiltonian for such a topological laser is described as

$$H = \begin{pmatrix} 2H_{\mathrm{QWZ}} & 2cI_2 & i\beta\sigma_x & 0 \\ 2cI_2 & 2H_{\mathrm{QWZ}} & 0 & i\beta'\sigma_x \\ i\beta\sigma_x & 0 & H^*_{\mathrm{QWZ}} & cI_2 \\ 0 & i\beta'\sigma_x & cI_2 & H^*_{\mathrm{QWZ}} \end{pmatrix}. \tag{19}$$

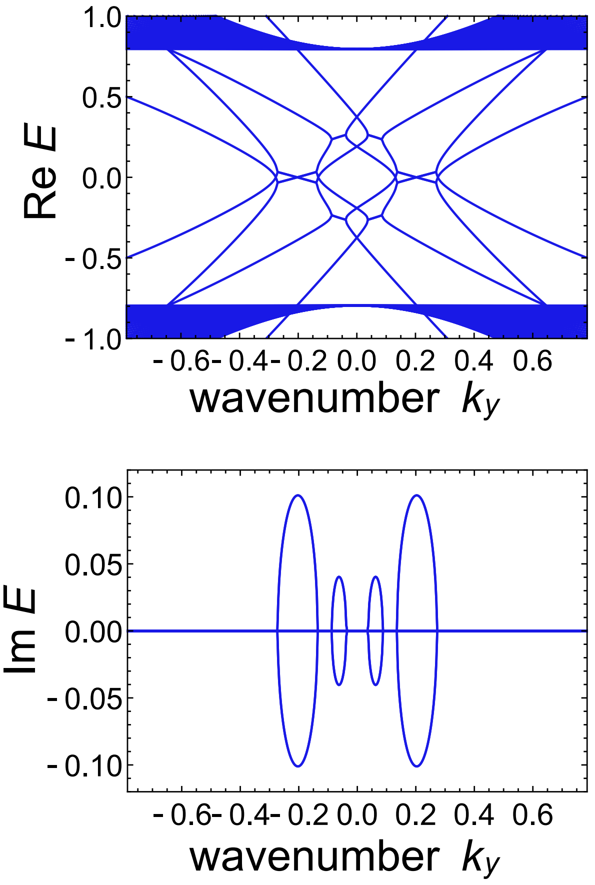

Supplementary Figure 3. **Lasing edge modes in the modified two-layered Bernevig-Hughes-Zhang model.** We arrange 50 sites in the x-direction with the open boundary and impose the periodic boundary condition in the y-direction. The parameters used are $u = -1$, $c = 0.2$, $\beta = 0.14$, and $\beta' = 0.06$.

As we constructed the topological insulator laser analyzed in the main text (cf. Eq. (5) and Fig. 4 in the main text), we modify the strength of the hopping in the two-layered QWZ model and couple the time-reversal two-layered QWZ model with a non-Hermitian coupling. Supplementary Figure 3 shows the edge band structure of this Hamiltonian. We can confirm the nonzero slope and the nonzero imaginary part of the energy in the energy dispersion corresponding to the lasing, mobile edge modes. We note that the model has the twice number of inner degrees of freedom compared to the topological insulator analyzed in the main text and thus exhibits more exceptional edge modes than in Fig. 4 in the main text.

Supplementary Note 7 - Exceptional edge modes in a continuum system

To show that continuum Hamiltonians can also exhibit exceptional edge modes, we construct a continuum model with the Hamiltonian

$$H = \begin{pmatrix} H_0 & C \\ -C^* & H_0^* \end{pmatrix}, \tag{20}$$

where H_0 is the continuum Hamiltonian for a Chern insulator with two gapless modes per edge:

$$H_0 = \begin{pmatrix} M - \beta\nabla^2 & a(-i\partial_x - \partial_y)^2 \\ a(-i\partial_x + \partial_y)^2 & -M + \beta\nabla^2 \end{pmatrix}, \tag{21}$$

and C is the non-Hermitian coupling:

$$C = \begin{pmatrix} 0 & ib \\ ib' & 0 \end{pmatrix} \tag{22}$$

with a, b, b', M, and β being real parameters. This model has the time-reversal symmetry in the same way as in the tight-binding model analyzed in the main text. By numerically diagonalizing the Hamiltonian, we obtain the edge band structure shown in Fig. 6 in the main text, where two gapless modes exist per edge, indicating the trivial bulk. However, those edge modes contain EPs and thus are stabilized against the disorder.

Supplementary Note 8 - Detail of the possible candidate of the proposed experimental setup of chiral active matter model

We expect that the exceptional edge modes can appear in chiral active matter using bacteria. In more detail, we consider bacteria, such as *E. coli*, swimming in the solution sandwiched by the upper and lower plates as shown in Supplementary Fig. 4. We set the distance between the two plates about 1 μm, i.e., slightly shorter than the bacteria length to realize both the chirality flipping and the interaction between bacteria with different chiralities. Chirality of the bacterial motion occurs due to the rotation of flagella and their interaction with the plate [14, 15]. Therefore, the direction of motion depends on which surface the bacteria are close to and can be flipped by shuttling between the upper and lower surfaces. For the clear chirality of the bacterial motion, the distance of two plates must be enough large. Previous research [15] has shown that the rotational movement disappears if the distance

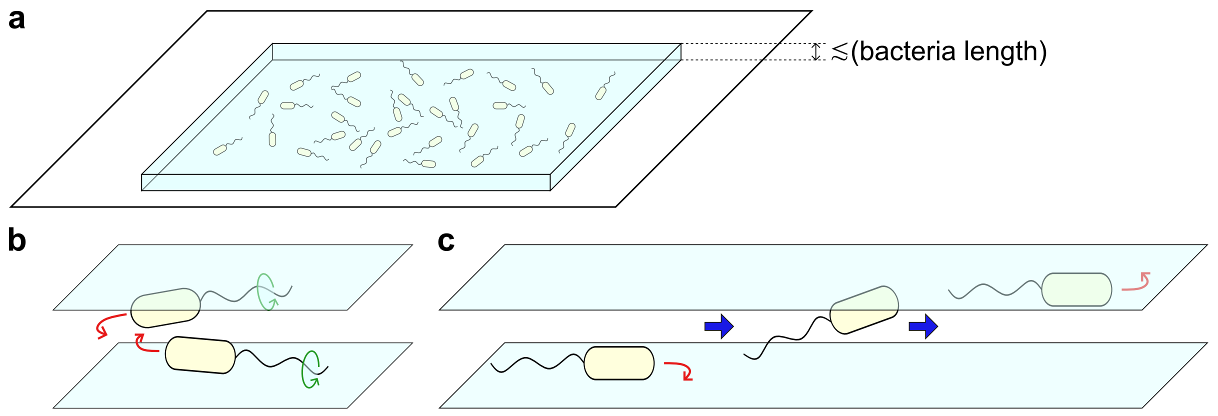

Supplementary Figure 4. **Possible experimental setup for the exceptional edge modes in chiral active matter. a,** Bacteria swimming between the upper and lower plates are considered. The distance between the two plates is set to be slightly shorter than the bacteria length. **b,** Bacteria swimming near the lower (upper) plates shows the clockwise (counterclockwise) motion due to the rotation of flagella when we see them from the upper side of the system. By the collision between two bacteria near the upper and lower surfaces, interaction occurs between chiral active particles with opposite chirality. **c,** The chirality of bacterial motion can flip by moving from the lower surface to the upper surface and vice versa.

between swimming bacteria and the surface plate becomes larger than a few tens percent of the length of the bacteria. Thus, we can expect that in the proposed system, one can observe the clear difference between the direction of rotation between the bacteria near the upper and lower surfaces.

On the other hand, aligning interaction between bacteria mainly originates from the collision of two bacteria. Since we set the distance between two plates shorter than the bacteria length, bacteria near the upper and lower surfaces can collide and interact with each other. One remaining problem is which type of interactions, nematic or polar interaction, dominates in the system. Previous research [16] has revealed that if the repulsion between two self-propelled rods like bacteria is dominant compared to the self-propulsion, a polar cluster tends to appear. Since our proposal is based on chiral active matter with polar interaction, exceptional edge modes may be more realizable when we suppress the activity of bacteria by, for example, controlling the concentration of the solution.

In our analysis, we consider the situation that anti-polar interaction emerges in hydrodynamics of active matter (see Supplementary Methods below for details), while it is not

obvious whether bacteria indeed exhibit such macroscopic anti-polar interaction or not in real experimental situations. As another candidate of chiral active matter that might show effective anti-polar interaction, we suggest the use of L-shaped active particles studied in previous research of chiral active matter [17]. L-shaped active particles can also lead to polar and/or anti-polar interaction and realize chirality flipping via the collision and the turnovers of the particles, thus possibly exhibiting exceptional edge modes.

Supplementary Methods - Derivation of the effective Hamiltonian for two-component chiral active matter

We describe the detailed derivation of the effective Hamiltonian for our active matter model via the Boltzmann-Ginzburg-Landau approach [18, 19]. We start with the particle model with polar (and anti-polar) interactions and rotational forces. We assume that the system is dilute such that three- and more-particle collisions are not significant. The dynamics of each particle is described as

$$\mathbf{r}_j(t+dt) = \mathbf{r}_j(t) + v_0 dt \mathbf{n}(\theta_j), \tag{23}$$

$$\theta_j(t+dt) = \begin{cases} \theta_j(t) + \omega^i dt + \xi_j & \text{(no confliction)}, \\ \Phi^{i,k}(\theta_j(t), \theta_l(t)) + \omega^i dt + \xi_j & \text{(when } j\text{th and } l\text{th particles conflict)}, \end{cases} \tag{24}$$

where $\mathbf{r}_j$ and θ_j are the location and the direction of the jth particle and i represents the direction of rotation. $\mathbf{n}(\theta_j)$ is the unit vector whose argument is θ_j. ω^i creates the constant rotational force and ξ_j is the Gaussian noise on the direction of each particle, which satisfies $\langle \xi_j(t) \rangle = 0$ and $\langle \xi_j(t)\xi_k(t') \rangle = T\delta_{jk}\delta(t-t')$ with T being the effective temperature. $\Phi^{i,k}$ is the angle after the collision between the i- and k-rotating particles, which can take, e.g., the mean value of the angles of two colliding particles to describe the polar interaction. Also, we assume that the chirality i flips occasionally at the rate γ. We note that if we consider $\omega^i = 0$ and only one component, this model reduces to the Vicsek model [20] except for the absence of the three- or more-particle confliction.

From the stochastic particle model, we derive the following Boltzmann equation,

$$\begin{aligned} &\partial_t f^i + v_0 \mathbf{n} \cdot \nabla f^i + \omega^i \partial_\theta f^i \\ &= D_0 \Delta f^i + D_1 g^{\alpha\beta} \partial_\alpha \partial_\beta f^i + I_{\mathrm{dif}}[f^i] + I_{\mathrm{col}}^{\mathrm{hom}}[f^i] + \sum_{j \neq i} I_{\mathrm{col}}^{\mathrm{het}}[f^i, f^j] + \sum_{j \neq i} \gamma (f^j - f^i), \end{aligned} \tag{25}$$

where f^i is the one-particle distribution function for the i-rotating particles and

$$I_{\text{dif}}[f^i] = -\lambda f^i + \lambda \int d\theta' \int d\eta P_\sigma(\eta)\delta_{m\pi}(\theta' + \eta - \theta) f^i(\mathbf{r}, \theta', t), \tag{26}$$

$$\begin{aligned} I_{\text{col}}^{\text{hom}}[f^i] &= -f^i(\mathbf{r}, \theta, t) \int d\theta' K(\theta' - \theta) f^i(\mathbf{r}, \theta', t) \\ &\quad + \int d\theta_1 \int d\theta_2 \int d\eta P_\sigma(\eta) K^i(\theta_2 - \theta_1) f^i(\mathbf{r}, \theta_1, t) f^i(\mathbf{r}, \theta_2, t) \delta_{m\pi}(\Phi^{i,i}(\theta_1, \theta_2) + \eta - \theta), \end{aligned} \tag{27}$$

$$\begin{aligned} I_{\text{col}}^{\text{het}}[f^i, f^j] &= -f^i(\mathbf{r}, \theta, t) \int d\theta' K(\theta' - \theta) f^j(\mathbf{r}, \theta', t) \\ &\quad + \int d\theta_1 \int d\theta_2 \int d\eta P_\sigma(\eta) K^{i,j}(\theta_2 - \theta_1) f^i(\mathbf{r}, \theta_1, t) f^j(\mathbf{r}, \theta_2, t) \delta_{m\pi}(\Phi^{i,j}(\theta_1, \theta_2) + \eta - \theta), \end{aligned} \tag{28}$$

are the self-diffusion integral, the collision integral between the same species, and that between the different species for each. $P(\eta)$ is the noise distribution, which is supposed to be Gaussian in the present setup, and $K(\theta_2 - \theta_1)$ represents the collision kernel. Here, we utilize the molecular chaos hypothesis, which assumes that the two-body distribution function can be described as the product of the one-body distribution functions.

To derive hydrodynamic equations, we conduct the Fourier transformation of the obtained Boltzmann equation. We consider the following Fourier(-like) components:

$$f_k^i(\mathbf{r}, t) = \int_{-\pi}^{\pi} f^i(\mathbf{r}, \theta, t) e^{ik\theta} d\theta, \tag{29}$$

$$P_k(\mathbf{r}, t) = \int_{-\pi}^{\pi} P(\eta) e^{ik\eta} d\eta, \tag{30}$$

$$I_{k,q}^i(\mathbf{r}, t) = \frac{1}{2\pi} \int_{-\pi}^{\pi} K^i(\Delta) e^{-iq\Delta + ik\Phi^{i,i}(0,\Delta)} d\Delta, \tag{31}$$

$$I_{k,q}^{i,j}(\mathbf{r}, t) = \frac{1}{2\pi} \int_{-\pi}^{\pi} K^{i,j}(\Delta) e^{-iq\Delta + ik\Phi^{i,j}(0,\Delta)} d\Delta. \tag{32}$$

It is noteworthy that one can calculate P_k, $I_{k,q}^i$, and $I_{k,q}^{i,j}$ from the microscopic parameters. Performing the Fourier transformation, we obtain the following equation:

$$\begin{aligned} &\frac{\partial f_k^i}{\partial t} + \frac{v_0}{2}(\nabla f_{k-1}^i + \nabla^* f_{k+1}^i) - ik\omega^i f_k^i \\ &= -\lambda(1 - P_k) f_k^i + D_0 \Delta f_k^i + \frac{D_1}{4}(\nabla^2 f_{k-2}^i + (\nabla^*)^2 f_{k+2}^i) \\ &\quad + \sum_{q=-\infty}^{\infty} (P_k I_{k,q}^i - I_{0,q}^i) f_q^i f_{k-q}^i + \sum_{j \neq i} \sum_{q=-\infty}^{\infty} (P_k I_{k,q}^{i,j} - I_{0,q}^{i,j}) f_q^i f_{k-q}^j + \sum_{j \neq i} \gamma(f_k^j - f_k^i), \end{aligned} \tag{33}$$

where $\nabla = \partial_x + i\partial_y$ and $\nabla^* = \partial_x - i\partial_y$. By definition, f_0^i and $(\mathrm{Re} f_1^i, \mathrm{Im} f_1^i)^T$ are equivalent to the density and the velocity field, respectively. With a little algebra, we obtain the equations for $k = 0, 1, 2$,

$$\frac{\partial \rho^i}{\partial t} + v_0 \mathrm{Re}(\nabla^* f_1^i) = D_0 \Delta \rho^i + \frac{D_1}{2} \mathrm{Re}(\nabla^{*2} f_2^i) + \sum_{j \neq i} \gamma(\rho^j - \rho^i), \tag{34}$$

$$\begin{aligned}
&\frac{\partial f_1^i}{\partial t} + \frac{v_0}{2}(\nabla \rho^i + \nabla^* f_2^i) - i\omega^i f_1^i \\
&= [\lambda(P_1 - 1) + (P_1 I_{1,0}^i - I_{0,0}^i + P_1 I_{1,1}^i - I_{0,1}^i)\rho^i] f_1^i + D_0 \Delta f_1^i + \frac{D_1}{4} \nabla^2 (f_1^i)^* \\
&\quad + \sum_{j \neq i} (P_1 I_{1,1}^{i,j} - I_{0,1}^{i,j}) \rho^j f_1^i + \sum_{j \neq i} (P_1 I_{1,1}^{i,j} - I_{0,1}^{i,j}) \rho^i f_1^j \\
&\quad + (P_1 I_{1,2}^i - I_{0,2}^i + P_1 I_{1,-1}^i - I_{0,-1}^i) f_2^i (f_1^i)^* \\
&\quad + \sum_{j \neq i} (P_1 I_{1,-1}^{i,j} - I_{0,-1}^{i,j}) f_2^j (f_1^i)^* + \sum_{j \neq i} (P_1 I_{1,2}^{i,j} - I_{0,2}^{i,j}) f_2^i (f_1^j)^* + \sum_{j \neq i} \gamma(f_1^j - f_1^i),
\end{aligned} \tag{35}$$

$$\begin{aligned}
&\frac{\partial f_2^i}{\partial t} + \frac{v_0}{2} \nabla f_2^i - 2i\omega^i f_2^i \\
&= \left(A^i + B^i \rho^i + \sum_{j \neq i} C^{i,j} \rho^j \right) f_2^i + \frac{D_1}{4} \nabla^2 \rho^i + (P_2 I_{2,1}^i - I_{0,1}^i)(f_1^i)^2 \\
&\quad + \sum_{j \neq i} (P_2 I_{2,1}^{i,j} - I_{0,1}^{i,j}) f_1^i f_1^j + \sum_{j \neq i} (P_2 I_{2,0}^{i,j} - I_{0,0}^{i,j}) \rho^i f_2^j + \sum_{j \neq i} \gamma(f_2^j - f_2^i),
\end{aligned} \tag{36}$$

where we omit the high-frequency terms, $f_{k'}^i$ $(k' \geq 3)$, which are irrelevant in the following discussion. Since we consider the polar active matter (that can have anti-polar interaction only between oppositely rotating particles), f_1^i becomes the leading order term. We have to balance the following terms in Supplementary Eqs. (34), (35), (36),

$$\partial_t \rho^i \sim \mathrm{Re} \nabla^* f_1^i, \quad \partial_t f_1^i \sim \nabla \rho^i, \quad f_2^i \sim (f_1^i)^2, \quad (f_1^i)^* f_2^i \sim \Delta f_1^i. \tag{37}$$

Therefore, we obtain the following scaling relations,

$$f_1^i \sim \epsilon, \quad f_2^i \sim \epsilon^2, \quad \rho^i - \rho_{\mathrm{ss}}^i \sim \epsilon, \quad \nabla \sim \epsilon, \quad \partial_t \sim \epsilon. \tag{38}$$

By considering the terms with the order ϵ^2, we can confirm that the f_2^i can be described as a linear combination of ∇f_1^j, $(f_1^j)^2$, and $f_1^j f_1^l$. By substituting this, we finally obtain the hydrodynamic equations,

$$\partial_t \rho^i + v_0 \nabla \cdot \mathbf{p}^i = D_0 \Delta \rho^i + \sum_j (\gamma \rho^j - \gamma \rho^i), \tag{39}$$

$$\begin{aligned}
\partial_t \mathbf{p}^i + \lambda(\mathbf{p}^i \cdot \nabla)\mathbf{p}^i &= \boldsymbol{\omega}^i \times \mathbf{p}^i + (a^i(\rho) + b^i(\mathbf{p}))\mathbf{p}^i + \sum_{j \neq i} (c^{i,j}(\rho) + d^{i,j}(\mathbf{p}))\mathbf{p}^j \\
&\quad - \mu^i \nabla \rho^i + D_0 \Delta \mathbf{p}^i + D_{\mathrm{odd}} \Delta \mathbf{p}^{i*} + (\text{higher-order terms}),
\end{aligned} \tag{40}$$

where i represents the chirality of active matter, i.e., the anticlockwise ($i = 1$) and clockwise ($i = 2$) moving direction. $\rho^i(\mathbf{r}, t)$ is the density field of active matter and $\mathbf{p}^i(\mathbf{r}, t) = \rho^i(\mathbf{r}, t)\mathbf{v}^i(\mathbf{r}, t)/v^i_{\rm ss}$ is the momentum field divided by the steady-state velocity, where $\mathbf{v}^i(\mathbf{r}, t)$ is the local average of velocities of self-propelled particles. Here, we omit some derivative terms and some higher-order terms with respect to ϵ. However, we remain the higher-order term including $\mathbf{p}^{i*} = (-p^i_y, p^i_x)^T$ to make the effective Hamiltonian compact (shown to be important for defining the topological invariant in continuum space [21]). This term is called odd viscosity and has been derived in the hydrodynamic equations of chiral active matter in previous research [22]. We note that these equations are similar to the Toner-Tu equations [23], hydrodynamic equations for one-component polar active matter. However, unlike the Toner-Tu equations, they contain a Coriolis-force-like term, $\boldsymbol{\omega}^i \times \mathbf{p}^i$, and momentum-coupling terms, $(c^{i,j}(\rho) + d^{i,j}(\mathbf{p}))\mathbf{p}^j$. The origins of the Coriolis-force-like term and the momentum-coupling terms are the chirality and the polar or anti-polar interaction between particles with the different chiralities. We note that there still remains the possibility of inconsistency between the polar or anti-polar interaction in the microscopic stochastic description and the hydrodynamic equations, because in the parameter region of the unordered phase, polar (or anti-polar) interaction does not lead to collective motion and instead can possibly enhance the anti-ordering with a help of the rotational motion of active matter. In the discussion below, we focus on the case that there is (effectively) anti-polar interaction in the hydrodynamic description between particles with opposite chiralities.

Linearizing the above hydrodynamic equations around a steady-state solution, we derive differential equations for the fluctuations of density and velocity fields,

$$\partial_t \delta\rho^i + \mathbf{v}^i_{\rm ss} \cdot \nabla\delta\rho^i = -\rho^i_{\rm ss}\nabla \cdot \delta\mathbf{v}^i + \sum_{j\neq i} \gamma(\rho^j - \rho^i), \tag{41}$$

$$\partial_t \delta\mathbf{v}^i + \lambda(\mathbf{v}^i_{\rm ss} \cdot \nabla)\delta\mathbf{v}^i = \boldsymbol{\omega}^i \times \delta\mathbf{v}^i + \alpha^i\delta\mathbf{v}^i + \sum_{j\neq i} \beta^{i,j}\delta\mathbf{v}^j - \mu^i\nabla\delta\rho^i + \nu^o\Delta\delta\mathbf{v}^{i*}, \tag{42}$$

where $\rho^i_{\rm ss}$ and $\mathbf{v}^i_{\rm ss}$ represent the steady-state values of the density and the velocity field, respectively, and $\delta\rho^i(\mathbf{r}, t) = \rho^i(\mathbf{r}, t) - \rho^i_{\rm ss}(\mathbf{r})$ and $\delta\mathbf{v}^i(\mathbf{r}, t) = \mathbf{v}^i(\mathbf{r}, t) - \mathbf{v}^i_{\rm ss}(\mathbf{r})$ are their fluctuations. While the above equations describe the linear dynamics around arbitrary steady-state solutions, here we consider the nonordered steady state, $\rho^1_{\rm ss} = \rho^2_{\rm ss} \equiv \rho_{\rm ss}$, $\mathbf{v}^i_{\rm ss} = 0$, and the symmetric or antisymmetric parameters, $c^{1,2}(\rho_{\rm ss}) + d^{1,2}(\mathbf{p}_{\rm ss}) = c^{2,1}(\rho_{\rm ss}) + d^{2,1}(\mathbf{p}_{\rm ss}) = a^i(\rho_{\rm ss}) + b^i(\mathbf{p}_{\rm ss}) \equiv -\beta$, $\omega^1 = -\omega^2 \equiv \omega_0$, $\rho^1_{\rm ss} = \rho^2_{\rm ss} \equiv \rho_0$, $\gamma^{1,2} = \gamma^{2,1} \equiv \gamma$. By nondimesion-

alizing the equations, we finally obtain the following linearized equations in the frequency domain,

$$\omega \begin{pmatrix} \delta\tilde{\rho}^1 \\ \delta\tilde{v}_x^1 \\ \delta\tilde{v}_y^1 \\ \delta\tilde{\rho}^2 \\ -\delta\tilde{v}_x^2 \\ -\delta\tilde{v}_y^2 \end{pmatrix} = H \begin{pmatrix} \delta\tilde{\rho}^1 \\ \delta\tilde{v}_x^1 \\ \delta\tilde{v}_y^1 \\ \delta\tilde{\rho}^2 \\ -\delta\tilde{v}_x^2 \\ -\delta\tilde{v}_y^2 \end{pmatrix} \tag{43}$$

with H being the effective Hamiltonian defined as

$$H = \begin{pmatrix} H_0 + A & C \\ -C^* & H_0^* + A \end{pmatrix}, \tag{44}$$

$$H_0 = \begin{pmatrix} 0 & -i\partial_x & -i\partial_y \\ -i\partial_x & 0 & -i(\omega_0 + \nu^o\Delta) \\ -i\partial_y & i(\omega_0 + \nu^o\Delta) & 0 \end{pmatrix}, \tag{45}$$

$$A = \begin{pmatrix} -i\gamma & 0 & 0 \\ 0 & -i\beta & 0 \\ 0 & 0 & -i\beta \end{pmatrix}. \tag{46}$$

$$C = \begin{pmatrix} i\gamma & 0 & 0 \\ 0 & i\beta & 0 \\ 0 & 0 & i\beta \end{pmatrix}, \tag{47}$$

where $\delta\tilde{\rho}^i$ and $\delta\tilde{v}_{x,y}^i$ represent the Fourier components of the nondimensionalized fluctuation of the density and the velocity field, respectively, and all the parameters are also nondimensionalized. We note that the Hamiltonian H_0 represents the effective Hamiltonian for topological fluid, which can be realized by utilizing chiral active matter [24]. Our Hamiltonian is no longer time-reversal symmetric. Since we combine the two topological systems with the opposite Chern numbers, the edge modes in Fig. 7b are not protected by the bulk topology. On the other hand, the coupling term C is non-Hermitian and thus creates EPs in the edge bands that protect the gapless modes. For numerical calculation, we use the parameters $\omega = 1$, $\nu = 0.5$, $\gamma = 0.3$, and $\beta = 0.5$.

While we have considered the two-valued rotational force ω, the distribution of rotational velocities must be continuous in practice. However, if we can separate moving particles into

two groups with clockwise and anticlockwise rotations, the coarse-grained dynamics of chiral active matter should not be changed. We can model such a situation by considering the following stochastic process of ω,

$$\frac{d\omega}{dt} = -\frac{\partial U}{\partial \omega} + \xi_\omega, \tag{48}$$

where U and ξ_ω represent the effective potential and the Gaussian noise, respectively. If we consider a double-well potential U that has minima at $\omega = \pm\omega_0$, the particles can be divided by their directions of rotations. We can derive the Boltzmann equation,

$$\begin{aligned} &\partial_t f + v_0 \mathbf{n} \cdot \nabla f + \omega \partial_\theta f \\ &= D_0 \Delta f + D_1 g^{\alpha\beta} \partial_\alpha \partial_\beta f + I_{\mathrm{dif}}[f] + I_{\mathrm{col}}[f] + \partial_\omega \left[\partial_\omega U + D_\omega \partial_\omega\right] f, \end{aligned} \tag{49}$$

where $f = f(\mathbf{r}, \theta, \omega, t)$ is the one-particle distribution function, and $I_{\mathrm{dif}}[f]$ and $I_{\mathrm{col}}[f]$ are the self-diffusion integral and the collision integral for each. By integrating the equation for $\omega < 0$ ($\omega > 0$), we can derive the time evolution of the one-particle distribution function for clockwise (anticlockwise) particles and check that the obtained equations are equivalent to the equations with the two-valued rotational force.

Supplementary Note 9 - Detail of the band structure of the active matter model

Here, we discuss the meaning of the crossings around the $\mathrm{Re}\, E = 0$ axis, which is not depicted as the red circle in Fig. 7 in the main text and what modifications of the edge dispersion are allowed. The crossings are the point where the unprotected edge dispersions with a diamond shape appear from the bulk dispersions around $\mathrm{Re}\, E = 0$. Therefore, it is impossible to separate the unstable edge dispersions with a diamond shape and the bulk dispersions around $\mathrm{Re}\, E = 0$. This constraint leads to the prohibition of moving the unstable edge dispersions to the upper or lower bulk dispersions as shown in Supplementary Fig. 5c. On the other hand, these edge dispersions are not protected by the topology or the EP, we can push them into the bulk dispersions around $\mathrm{Re}\, E = 0$ depicted in Supplementary Fig. 5a. Besides, the crossings in the bulk gap are not true degeneracies since the edge bands have different imaginary parts of the eigenfrequencies and thus can be removed by moving the unstable edge dispersions to the right or left as demonstrated in Supplementary Fig. 5b.

Supplementary Note 10 - Bernard-LeClair symmetry class of our models

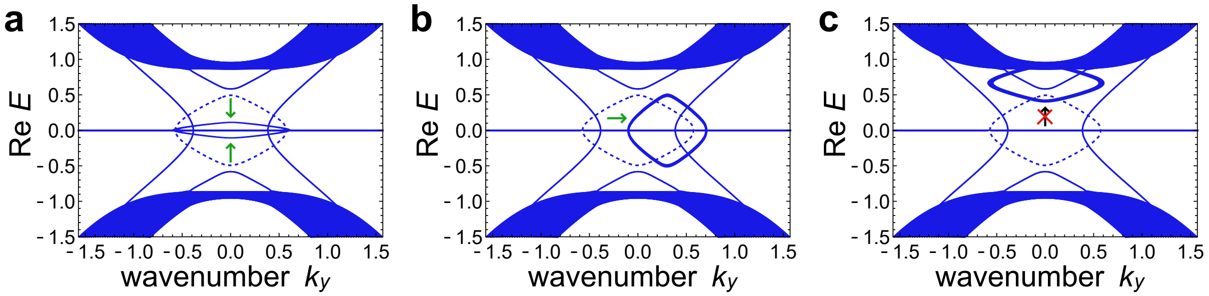

Supplementary Figure 5. **Allowed and prohibited modifications of the dispersion relation in the chiral active matter model. a-c,** Each figure shows what modification of the dispersion relation with a diamond shape in Fig. 7 in the main text is allowed (the green arrows) or prohibited (the black arrow with the red cross). **a,** We can push the edge dispersion into the bulk dispersion around the $\mathrm{Re}\, E = 0$ axis. **b,** Moving the edge dispersion to the right is also possible. In this case, the apparent crossing disappears. **c,** Pushing up the edge dispersion to the upper bulk dispersion is prohibited.

In Hermitian systems, three types of $\mathbb{Z}_2$ symmetries (i.e., the time-reversal symmetry, the particle-hole symmetry, and the chiral (sublattice) symmetry) play an important role in the classification of topological band structures [25, 26]. The time-reversal (particle-hole, chiral) symmetry means that there exists a unitary operator T (C, Γ) which satisfies $TH(\mathbf{k})T^{-1} = H(-\mathbf{k})^*$ ($CH(\mathbf{k})C^{-1} = -H(-\mathbf{k})^T$, $\Gamma H(\mathbf{k})\Gamma^{-1} = -H(\mathbf{k})^\dagger$) and $TT^* = \pm 1$ ($CC^* = \pm 1$, $\Gamma^2 = 1$), where $H(\mathbf{k})$ is the Bloch Hamiltonian. Because of $\Gamma = CT$, if there are two of these symmetries, the system also exhibits the other $\mathbb{Z}_2$ symmetry. Therefore, we can classify Hermitian Hamiltonians into 10 Altland-Zirnbauer (AZ) classes [26] concerning the $\mathbb{Z}_2$ symmetries.

Recent researches [9, 10, 27] have extended the $\mathbb{Z}_2$ symmetries and the AZ symmetry classes to non-Hermitian systems. We need to reconsider the discrepancy between H and $H^\dagger$ and thus distinguish two types of each $\mathbb{Z}_2$ symmetry, i.e., $TH(\mathbf{k})T^{-1} = H(-\mathbf{k})^*$ and $TH(\mathbf{k})T^{-1} = H(-\mathbf{k})^T$ for the time-reversal symmetry, $CH(\mathbf{k})C^{-1} = -H(-\mathbf{k})^T$ and $CH(\mathbf{k})C^{-1} = -H(-\mathbf{k})^*$ for the particle-hole symmetry, $\Gamma H(\mathbf{k})\Gamma^{-1} = -H(\mathbf{k})^\dagger$ and $\Gamma H(\mathbf{k})\Gamma^{-1} = -H(\mathbf{k})$ for the chiral (sublattice) symmetry. In a previous paper [10], the symmetry $\Gamma H(\mathbf{k})\Gamma^{-1} = -H(\mathbf{k})^\dagger$ is called the chiral symmetry, and $\Gamma H(\mathbf{k})\Gamma^{-1} = -H(\mathbf{k})$ is called the sublattice symmetry, and the two other $\mathbb{Z}_2$ symmetries are distinguished by

adding the dagger for the latter definition. For the topological classification, it is evident that the daggered time-reversal symmetry (the daggered particle-hole symmetry) coincides with the particle-hole symmetry (the time-reversal symmetry). By utilizing these extended $\mathbb{Z}_2$ symmetries, one can obtain the 38 Bernard-LeClair (BL) classes [10, 27–29]. In non-Hermitian systems, the chiral symmetry and the sublattice symmetry are independent of each other, and thus increase the number of the symmetry classes. In particular, whether the sublattice-symmetry operator commutes or anticommutes with each of the other $\mathbb{Z}_2$ operators plays an important role in the classification.

The periodic table for topological insulators predicts the existence or absence of topological invariants for the Bloch Hamiltonian in each AZ class. To define the topological index in non-Hermitian Hamiltonians, we must reconsider the energy gap. In Hermitian systems, the eigenenergies distribute on the one-dimensional energy axis and thus have only point-like gaps in their spectra. On the contrary, in non-Hermitian systems, the eigenenergies can be complex and thus distribute on the two-dimensional space, which leads to the variety of the definitions of the energy gaps [9]. Especially, the periodic table for non-Hermitian

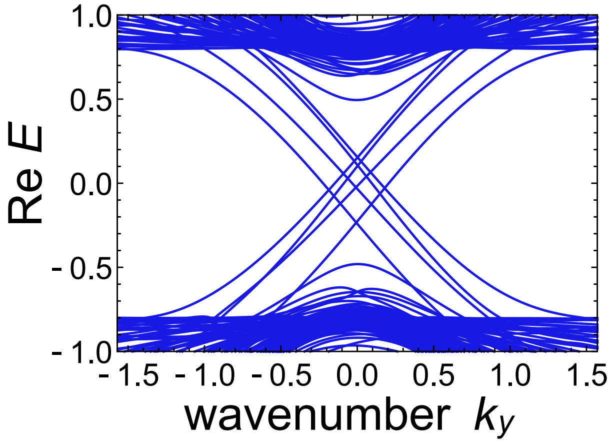

Supplementary Figure 6. **Robust gapless edge modes in the S_+AII class.** We calculate the edge band structure of the two-layered non-Hermitian Bernevig-Hughes-Zhang model with the on-site imaginary potential and the complex noise in the non-Hermitian coupling and without the Hermitian coupling. We align 50 sites in the x direction with the open boundary and impose the periodic boundary condition in the y direction. The parameters used are $u = -1$, $c = 0.2$, $\beta = 0.14$, $\beta' = 0.06$, $\gamma = 0$, and $g = 1.1$. The noise width for the real noise in the non-Hermitian coupling is $W = 1.0$.

Hamiltonians deals with line gaps and point gaps. If we can draw a line that separates complex energy spectra, the band structures have the line gap. On the other hand, energy spectra can encircle a point in the complex energy space, which never occurs in Hermitian Hamiltonians. Such a point is called a point gap and leads to topological invariants unique to non-Hermitian systems. Besides, to properly take the constraint of the symmetry into account, we need to consider both the line gaps parallel to the real and imaginary axes of the complex energy space for some BL classes. The periodic table indicates what type of topological invariants appear for each kind of energy gap in the Hamiltonian belonging to each non-Hermitian BL class.

One can discuss the symmetries and the topological invariants in the Hamiltonians of our models. Here, we focus on the lattice model with the on-site imaginary potential (cf. Fig. 3d in the main text) and without the Hermitian coupling $\gamma\sigma_x \otimes \sigma_y \otimes \sigma_x$. As in Fig. 3d in the main text, this model exhibits robust gapless edge modes shown in Supplementary Fig. 6. However, these gapless edge modes are not characterized by the topological invariant predicted from the periodic table [9, 10, 27]. The Hamiltonian is described as follows in terms of the Pauli matrices σ_i,

$$\begin{aligned} H(\mathbf{k}) = {} & (u + \cos k_x + \cos k_y) I_2 \otimes I_2 \otimes \sigma_z + \sin k_y I_2 \otimes I_2 \otimes \sigma_y + \sin k_x \sigma_z \otimes I_2 \otimes \sigma_x \\ & + c I_2 \otimes \sigma_x \otimes I_2 + i\frac{\beta + \beta'}{2} \sigma_x \otimes I_2 \otimes \sigma_x \\ & + i\frac{\beta - \beta'}{2} \sigma_x \otimes \sigma_z \otimes \sigma_x + ig\sigma_z \otimes I_2 \otimes I_2. \end{aligned} \tag{50}$$

From this expression and the anticommutation relations of the Pauli matrices, we can confirm that the following unitary operators represent the $\mathbb{Z}_2$ symmetries of the Hamiltonian:

$$T = (i\sigma_y) \otimes I_2 \otimes I_2, \quad TH(\mathbf{k})T^{-1} = H^*(-\mathbf{k}) \quad \text{(time reversal symmetry)}, \tag{51}$$

$$S = (i\sigma_y) \otimes \sigma_z \otimes \sigma_x, \quad SH(\mathbf{k})S^{-1} = -H(\mathbf{k}) \quad \text{(sublattice symmetry)}. \tag{52}$$

Furthermore, these operators satisfy $TT^* = -1$ and $[T, S] = 0$. Therefore, we conclude that this Hamiltonian belongs to the S_+AII class according to a proposed periodic table [10] and thus should have only the trivial index. However, as shown in Supplementary Fig. 6, this Hamiltonian can exhibit robust gapless edge modes, which cannot be captured by the periodic table, thus demonstrating the breakdown of the bulk-edge correspondence. While one can readily check that the other Hamiltonians can have nontrivial bulk indices, exceptional edge modes in those models also can emerge independently of the bulk topological

invariants. Thus, the predictions from the periodic table are irrelevant to the presence or absence of robust gapless edge modes proposed in the present work.

Supplementary Note 11 - $\mathbb{Z}_2$ symmetries in our models and their roles in the protection of exceptional edge modes

Exceptional points in exceptional edge modes can be protected by the topology associated with the branchpoint singularity and the symmetries, such as the PT symmetry, the CP symmetry, the chiral symmetry, and the pseudo-Hermiticity [30] as discussed in the main text. To confirm this point, we describe the $\mathbb{Z}_2$ symmetries of our models related to the protection of EPs. The symmetry in the bulk system should agree with that in the edge and thus should determine whether the EPs can be topologically protected or not. However, we find that the conventional PT, CP symmetries, and the modified pseudo-Hermiticity play no roles in determining the protection of exceptional edge modes, as discussed in the following paragraph.

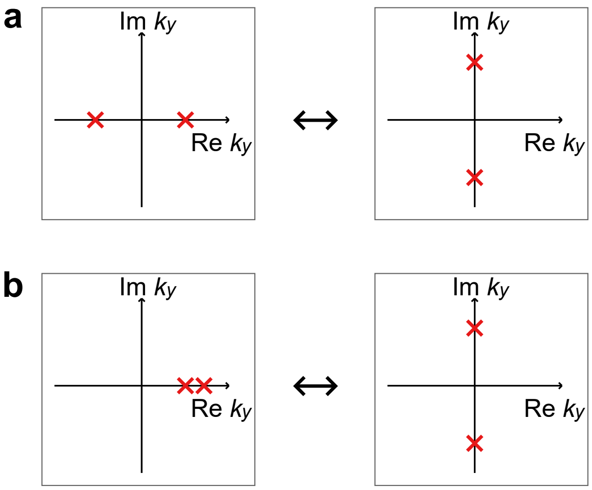

Supplementary Figure 7. **Eigenvalues of edge modes around EPs. a-b,** Eigenvalues of edge modes around the EP are presented. **a,** The eigenvalues of the two edge modes localized at the same side are changed from a pair of opposite real values to complex conjugates around the EP, for example in Fig. 1b around the $\mathrm{Re}\, E = 0$ axis. **b,** The eigenvalues of the edge modes localized at the opposite sides have the same eigenvalues before crossing the EP. The edge modes considered here are converted into each other by applying the conventional PT, CP, or modified pseudo-Hermitian operation.

First, we confirm the symmetries of the two-layered non-Hermitian Bernevig-Hughes-Zhang model. It has the PT symmetry and the pseudo-Hermiticity defined as,

$$PT = \sigma_x \otimes I_2 \otimes \sigma_z,\ PTH(\mathbf{k})(PT)^{-1} = H^*(\mathbf{k}), \tag{53}$$

$$\Gamma = \sigma_x \otimes \sigma_z \otimes \sigma_x,\ \Gamma H(\mathbf{k})\Gamma^{-1} = -H^\dagger(\mathbf{k}). \tag{54}$$

Furthermore, we can also define the following modified PT symmetry:

$$P'T = I_2 \otimes I_2 \otimes \sigma_z,\ P'TH(k_x,k_y)(P'T)^{-1} = H^*(-k_x,k_y), \tag{55}$$

$$P''T = \sigma_z \otimes I_2 \otimes I_2,\ P''TH(k_x,k_y)(P''T)^{-1} = H^*(k_x,-k_y). \tag{56}$$

The model without the Hermitian coupling ($\gamma = 0$) also has the conventional and modified CP symmetry and pseudo-Hermiticity defined as,

$$CP = \sigma_z \otimes \sigma_z \otimes \sigma_y,\ CPH(\mathbf{k})(CP)^{-1} = -H^*(\mathbf{k}), \tag{57}$$

$$CP' = \sigma_y \otimes \sigma_z \otimes \sigma_y,\ CP'H(k_x,k_y)(CP')^{-1} = -H^*(-k_x,k_y), \tag{58}$$

$$CP'' = \sigma_x \otimes \sigma_z \otimes \sigma_x,\ CP''H(k_x,k_y)(CP'')^{-1} = -H^*(k_x,-k_y), \tag{59}$$

$$\eta = \sigma_z \otimes I_2 \otimes I_2,\ \eta H(\mathbf{k})\eta^{-1} = H^\dagger(\mathbf{k}). \tag{60}$$

$$\eta' = \sigma_y \otimes I_2 \otimes I_2,\ \eta' H(k_x,k_y)(\eta')^{-1} = H^\dagger(-k_x,k_y), \tag{61}$$

$$\eta'' = \sigma_x \otimes I_2 \otimes \sigma_z,\ \eta'' H(k_x,k_y)(\eta'')^{-1} = H^\dagger(k_x,-k_y). \tag{62}$$

The modified symmetries play a similar role as in the conventional counterpart under the open boundary condition in the x or y direction. For example, the modified symmetry defined by $P'T$ leads to the PT symmetry under the open boundary condition in the x direction because the wavenumber k_x is no longer a good quantum number in this situation. The random real on-site potential and the imaginary noise in the non-Hermitian coupling (cf. Methods) only satisfy the modified PT symmetry defined by $P'T$ and $P''T$. The exceptional edge modes are robust against these disorders (cf. Fig. 3b in the main text), which implies that the modified PT symmetry acts as the PT symmetry in the effective edge Hamiltonian (cf. Eq. (3) in the main text) and thus can protect the exceptional edge modes. On the contrary, the real noise in the non-Hermitian coupling considered in Fig. 3c in the main text breaks all the symmetries above and thus opens a gap in the edge bands. We can also check that the imaginary on-site potential considered to calculate the band structures in Fig. 3d in the main text and in Supplementary Fig. 6 preserves the conventional PT

and CP symmetries and the modified pseudo-Hermiticity. However, this additional term removes the EPs from the edge modes, which is against the naive expectation from the discussion using the symmetry of the effective edge Hamiltonian. We explain the reason in the next paragraph.

The disappearance of exceptional edge modes from the band structures in Fig. 3d in the main text and in Supplementary Fig. 6 indicates that the conventional PT and CP symmetries and the modified pseudo-Hermiticity play no roles in protecting the exceptional edge modes. We can explain this from the fact that the operators, PT, CP, η', and η'', convert an edge mode into another edge mode localized at the opposite side of the system. Thus, the 2×2 effective Hamiltonian (cf. Eq. (3) in the main text) is insufficient to explain what constraint these symmetries impose on the edge modes because it only considers the edge modes localized at one side of the system. To deal with the edge modes at both sides simultaneously, we need a 4×4 effective edge Hamiltonian. For both the 2×2 and 4×4 effective edge Hamiltonian, we can calculate the following topological invariant[9, 30],

$$\nu = \mathrm{sgn}(\det H(k_0 - \delta) H(k_0 + \delta)) \tag{63}$$

which guarantees the robustness of EPs in one-dimensional systems. Here, k_0 is the wavenumber at the EP, and δ is a small real number. We confirm that the topological invariant for the 2×2 effective edge Hamiltonian is nontrivial, $\nu = -1$, while that for the 4×4 effective edge Hamiltonian is trivial, $\nu = 1$. Supplementary Figure 7 shows the change of the eigenvalues around the EP and schematically explains the triviality and nontriviality of the topological invariants. Crossing the EP, the two eigenvalues of exceptional edge modes localized at the same side becomes a pair of complex conjugates from a pair of opposite real numbers or vice versa. On the contrary, the eigenvalues of two edge modes which are transformed into each other by the operators, PT, CP, η', and η'', take the same real value in a certain range of the wavenumber. Calculating the topological invariant from these eigenvalues, we can confirm its nontriviality (triviality) for the former (latter) case, corresponding to the 2×2 (4×4) effective edge Hamiltonian. Therefore, the conventional PT symmetry, the conventional CP symmetry, the modified pseudo-Hermiticity are irrelevant to the protection of exceptional edge modes, while the modified PT symmetry, the modified CP symmetry, the conventional pseudo-Hermiticity, and the chiral symmetry can protect the exceptional edge modes.

Finally, we show the symmetries of other models and that there exist symmetries that can protect the EPs in the edge bands. In the same manner as in the trivial tight-binding model, the nontrivial tight-binding model (cf. Eq. (2) in the main text and Supplementary Eq. (9)) exhibits the PT symmetry, the CP symmetry, the chiral symmetry, and the pseudo-Hermiticity defined as,

$$PT = \sigma_x \otimes \sigma_z,\ PTH(\mathbf{k})(PT)^{-1} = H^*(\mathbf{k}), \tag{64}$$

$$CP = \sigma_z \otimes \sigma_y,\ CPH(\mathbf{k})(CP)^{-1} = -H^*(\mathbf{k}), \tag{65}$$

$$\Gamma = \sigma_x \otimes \sigma_x,\ \Gamma H(\mathbf{k})\Gamma^{-1} = -H^\dagger(\mathbf{k}), \tag{66}$$

$$\eta = \sigma_z \otimes I_2,\ \eta H(\mathbf{k})\eta^{-1} = H^\dagger(\mathbf{k}), \tag{67}$$

and the modified PT symmetry, CP symmetry, and pseudo-Hermiticity:

$$P'T = I_2 \otimes \sigma_z,\ P'TH(k_x, k_y)(P'T)^{-1} = H^*(-k_x, k_y), \tag{68}$$

$$P''T = \sigma_z \otimes I_2,\ P''TH(k_x, k_y)(P''T)^{-1} = H^*(k_x, -k_y), \tag{69}$$

$$CP' = \sigma_y \otimes \sigma_y,\ CP'H(k_x, k_y)(CP')^{-1} = -H^*(-k_x, k_y), \tag{70}$$

$$CP'' = \sigma_x \otimes \sigma_x,\ CP''H(k_x, k_y)(CP'')^{-1} = -H^*(k_x, -k_y), \tag{71}$$

$$\eta' = \sigma_y \otimes I_2,\ \eta' H(k_x, k_y)(\eta')^{-1} = H^\dagger(-k_x, k_y), \tag{72}$$

$$\eta'' = \sigma_x \otimes \sigma_z,\ \eta'' H(k_x, k_y)(\eta'')^{-1} = H^\dagger(k_x, -k_y). \tag{73}$$

Again, the conventional PT and CP symmetry, and the modified pseudo-Hermiticity play no roles in protecting exceptional edge modes, which we can confirm from the result in Supplementary Fig. 2d. The topological laser analyzed in Fig. 4 and 5 in the main text has only the following modified PT symmetry,

$$P'T = I_2 \otimes \sigma_z,\ P'TH(k_x, k_y)(P'T)^{-1} = H^*(-k_x, k_y), \tag{74}$$

$$P''T = \sigma_z \otimes I_2,\ P''TH(k_x, k_y)(P''T)^{-1} = H^*(k_x, -k_y), \tag{75}$$

as symmetries related to the protection of exceptional edge modes. The continuous model analyzed in Fig. 6 in the main text is rewritten as

$$\begin{aligned} H(\mathbf{k}) = {} & [M + \beta(k_x^2 + k_y^2)] I_2 \otimes \sigma_z + a(k_x^2 - k_y^2) I_2 \otimes \sigma_x \\ & + 2a k_x k_y \sigma_z \otimes \sigma_y + i\frac{b+b'}{2}\sigma_x \otimes \sigma_x + \frac{-b+b'}{2}\sigma_x \otimes \sigma_y. \end{aligned} \tag{76}$$

We can confirm that this model exhibits the modified PT symmetry and the chiral symmetry defined as,

$$P'T = \sigma_z \otimes I_2,\ P'TH(k_x, k_y)(P'T)^{-1} = H^*(-k_x, k_y), \tag{77}$$

$$P''T = \sigma_z \otimes I_2,\ P''TH(k_x, k_y)(P''T)^{-1} = H^*(k_x, -k_y), \tag{78}$$

$$\Gamma = \sigma_y \otimes \sigma_y,\ \Gamma H(\mathbf{k})\Gamma^{-1} = -H^\dagger(\mathbf{k}). \tag{79}$$

Finally, the effective Hamiltonian of the chiral active matter model only shows the chiral symmetry

$$\Gamma = \sigma_x \otimes I_3,\ \Gamma H(\mathbf{k})\Gamma^{-1} = -H^\dagger(\mathbf{k}), \tag{80}$$

where I_3 is a 3×3 identity matrix.

Supplementary Note 12 - Exceptional edge modes protected by the chiral symmetry

Since EPs can be protected by the chiral symmetry and the topology of a system, exceptional edge modes can also exist robustly under the chiral symmetry. To confirm the robustness of exceptional edge modes against chiral-symmetry-preserving disorder, we consider the two-layered non-Hermitian Bernevig-Hughes-Zhang model with the imaginary noise in the non-Hermitian coupling considered in Fig. 3 in the main text and disorder $id(x)\sigma_x \otimes I \otimes I$. Here, $d(x)$ takes a random real value for each x from uniform distributions ranging $[-w, w] \in \mathbb{R}$. We use $w = 0.05$, and the other parameters are the same as used in Fig. 3 in the main text. These disorders break the conventional and modified PT, CP symmetries, and pseudo-Hermiticity. Therefore, the chiral symmetry, $\Gamma = \sigma_x \otimes \sigma_z \otimes \sigma_x$, $\Gamma H(\mathbf{k})\Gamma^{-1} = -H^\dagger(\mathbf{k})$, is the only symmetry that can protect the exceptional edge modes in this disordered system. Supplementary Figure 8 shows the edge band structure under the disorders. The EPs remain and protect the existence of edge modes, which implies that the chiral symmetry can also support the robustness of exceptional edge modes. We note that the eigenvalues do not appear as real values or pairs of complex conjugates, while the PT symmetry and the pseudo-Hermiticity lead to such constraint on the band structure. Since the chiral symmetry is the sole symmetry that can protect the exceptional edge modes in the active matter model analyzed in Fig. 7 in the main text as discussed in the previous section, this result also guarantees the robustness of exceptional edge modes there.

We can also confirm that this model is topologically trivial in the conventional sense and thus clearly breaks the bulk-edge correspondence. The model belongs to AIII class, which only has the chiral symmetry and thus can only exhibit a trivial invariant for a real line gap [10, 27]. The topological invariant for an imaginary line gap cannot be defined because the model has no imaginary line gaps. The topological invariant for a point gap in AIII class systems can be defined by the Chern number of $iH\Gamma$ [30] (note that $iH\Gamma$ is Hermitian and if it has a zero-energy eigenstate ψ, $\Gamma\psi$ is a zero-energy eigenstate of the non-Hermitian Hamiltonian H). We numerically confirm the triviality of the topological invariant for the

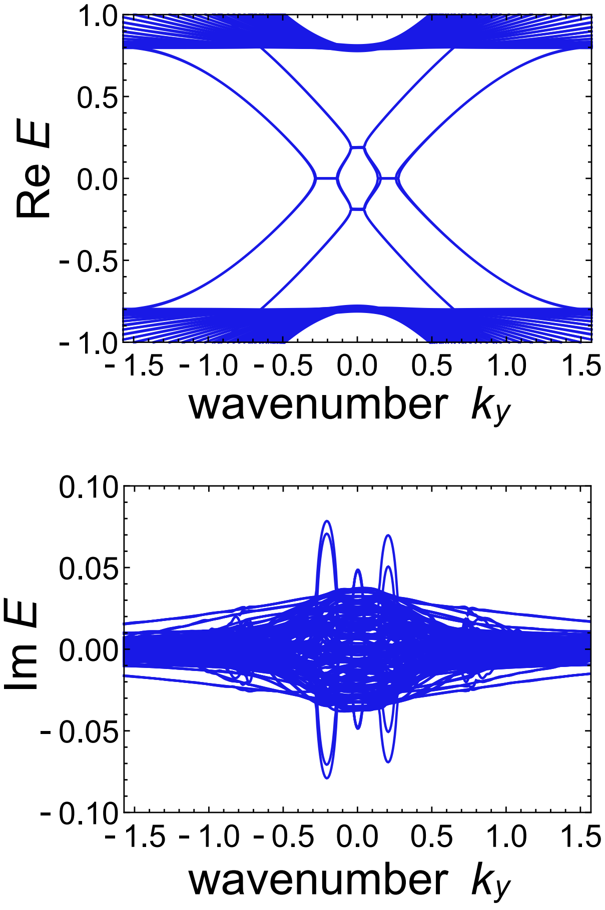

Supplementary Figure 8. **Exceptional edge modes under chiral-symmetry-preserving disorder.** We arrange 50 sites in the x direction with the open boundary and impose the periodic boundary condition in the y direction. The parameters used are $u = -1$, $c = 0.2$, $\beta = 0.14$, $\beta' = 0.06$ and $\gamma = 0.05$. The noise width is $w = 0.05$, $(W = 0.05)$ for the disorder $id(x)\sigma_x \otimes I \times I$ (the imaginary noise in the non-Hermitian coupling).

present model with constant $d(x) = 0.05$ and without the real noise in the non-Hermitian coupling by using the numerical methods proposed by Fukui, T., Hatsugai, Y., and Suzuki, H. [31]. Here we set the other parameters to be the same as in Supplementary Fig. 8. Therefore, the model analyzed here is topologically trivial in the conventional sense, while it exhibits robust exceptional edge modes. Since the model exhibits no spatial symmetries like the PT symmetry, the bulk-edge correspondence should indicate the consistency between the topological invariant predicted from the periodic table [9, 10, 27] and the existence of the robust edge modes, which is broken in the present model.

Supplementary References

[1] Qi X. L., Wu Y. S., & Zhang S. C. Topological quantization of the spin Hall effect in two-dimensional paramagnetic semiconductors. *Phys. Rev. B* **74,** 085308 (2006).

[2] Trescher, M. & Bergholtz, E. J. Flat bands with higher Chern number in pyrochlore slabs. *Phys. Rev. B* **86,** 241111 (2012).

[3] Liu, Z., Bergholtz, E. J., Fan, H., & Läuchli, A. M. Fractional Chern Insulators in Topological Flat Bands with Higher Chern Number. *Phys. Rev. Lett.* **109,** 186805 (2012).

[4] Kunst, F. K., Edvardsson, E., Budich, J. C., & Bergholtz, E. J. Biorthogonal Bulk-Boundary Correspondence in Non-Hermitian Systems. *Phys. Rev. Lett.* **121,** 026808 (2018).

[5] Xiong, Y. Why does bulk boundary correspondence fail in some non-hermitian topological models. *J. Phys. Commun.* **2,** 035043 (2018).

[6] Yao, S. & Wang, Z. Edge States and Topological Invariants of Non-Hermitian Systems. *Phys. Rev. Lett.* **121,** 086803 (2018).

[7] Lee, C. H. & Thomale, R. Anatomy of skin modes and topology in non-Hermitian systems. *Phys. Rev. B* **99,** 201103 (2019).

[8] Yokomizo, K. & Murakami, S. Non-Bloch Band Theory of Non-Hermitian Systems. *Phys. Rev. Lett.* **123,** 066404 (2019).

[9] Gong, Z. et al., Topological Phases of Non-Hermitian Systems. *Phys. Rev. X* **8,** 031079 (2018).

[10] Kawabata K., Shiozaki K., Ueda M., & Sato M. Symmetry and Topology in Non-Hermitian Physics. *Phys. Rev. X* **9,** 041015 (2019).

[11] Shen, H., Zhen, B., & Fu, L. Topological Band Theory for Non-Hermitian Hamiltonians. *Phys. Rev. Lett.* **120,** 146402 (2018).

[12] Harari, G. et al., Topological insulator laser: Theory. *Science* **359,** 1230? (2018).

[13] Bandres, M. A. et al., Topological insulator laser: Experiments. *Science* **359,** 1231? (2018).

[14] DiLuzio, W. R. et al., Escherichia coli swim on the right-hand side. *Nature* **435,** 1271–1274 (2005).

[15] Li, G., Tam, L. K., & Tang, J. X. Amplified effect of Brownian motion in bacterial near-surface swimming. *Proc. Natl. Acad. Sci. USA* **105,** 18355–18359 (2008).

[16] Bär, M., Großmann, R., Heidenreich, S., & Peruani, F., Self-Propelled Rods: Insights and Perspectives for Active Matter. *Annu. Rev. Condens. Matter. Phys.* **11,** 441–466 (2020).

[17] Kümmel, F. et al., Circular Motion of Asymmetric Self-Propelling Particles. *Phys. Rev. Lett.* **110,** 198302 (2013).

[18] Bertin, E., Droz, M., & Grégoire, G. Boltzmann and hydrodynamic description for self-propelled particles. *Phys. Rev. E* **74,** 022101 (2006).

[19] Peshkov, A., Bertin, E., Ginelli, F., & Chaté, H. Boltzmann-Ginzburg-Landau approach for continuous descriptions of generic Vicsek-like models. *Eur. Phys. J. Special Topics* **223,** 1315–1344 (2014).

[20] Vicsek, T., Czirók A., Ben-Jacob, E., Cohen, I., & Shochet, O. Novel Type of Phase Transition in a System of Self-Driven Particles. *Phys. Rev. Lett.* **75,** 1226 (1995).

[21] Bal, G. Continuous bulk and interface description of topological insulators. *J. Math. Phys.* **60,** 081506 (2019).

[22] Banerjee, D., Souslov, A., Abanov, A. G., & Vitelli, V. Odd viscosity in chiral active fluids. *Nat. Commun.* **8,** 1573 (2017).

[23] Toner, J. & Tu, Y. Long-Range Order in a Two-Dimensional Dynamics XY model: How Birds Fly Together. *Phys. Rev. Lett.* **75,** 4326 (1995).

[24] Souslov, A., Dasbiswas, K., Fruchart, M., Vaikuntanathan, S., & Vitelli, V. Topological Waves in Fluids with Odd Viscosity. *Phys. Rev. Lett.* **122,** 128001 (2019).

[25] Altland, A. & Zirnbauer, M. R. Nonstandard Symmetry Classes in Mesoscopic Normal-Superconducting Hybrid Structures. *Phys. Rev. B* **55,** 1142 (1997).

[26] Hazan, M. Z. & Kane, C. L. Colloquium: Topological insulators. *Rev. Mod. Phys.* **82,** 3045–3067 (2010).

[27] Zhou, H. & Lee, J. Y. Periodic table for topological bands with non-Hermitian symmetries. *Phys. Rev. B* **99,** 235112 (2019).

[28] Bernard, D. & LeClair, A. A Classification of Non-Hermitian Random Matrices. in *Statistical Field Theories* (eds. Cappelli, A. & Mussardo G.) 207-214 (Springer, Dordrecht, 2002).

[29] Bernard, D. & LeClair, A. A classification of 2D random Dirac fermions. *J. Phys. A: Math. Gen.* **35**, 2555-2567 (2002).

[30] Kawabata, K., Bessho, T., & Sato M. Classification of Exceptional Points and Non-Hermitian Topological Semimetals. *Phys. Rev. Lett.* **123,** 066405 (2019).

[31] Fukui, T., Hatsugai, Y., & Suzuki, H. Chern Numbers in Discretized Brillouin Zone: Efficient Method of Computing (Spin) Hall Conductances. *J. Phys. Soc. of Jpn.* **74,** 1674–1677 (2005).
