# Supplementary material for: Exceptional non-Hermitian topological edge mode and its application to active matter
Source: Nat Commun. 2020 Nov 12;11:5745. doi: 10.1038/s41467-020-19488-0 (PMC7665040; doi:10.1038/s41467-020-19488-0)
Supplement: Supplementary file 3 — Description of Additional Supplementary Files [file 41467_2020_19488_MOESM3_ESM.pdf]

## Description of Additional Supplementary Files

Supplementary Movie 1. Real-space simulation of the topological laser system without the non-Hermitian coupling. The color represents the probability amplitude at each site, which is normalized so that the sum of squares gives unity. No lasing edge modes are observed.

Supplementary Movie 2. Real-space simulation of the lasing edge modes. The color represents the probability amplitude at each site, which is normalized so that the sum of squares gives unity.

Supplementary Movie 3. Real-space simulation of the lasing edge modes propagating along the edge of the sample. The color represents the probability amplitude at each site, which is normalized so that the sum of squares gives unity. The grey circles represent the absence of the sites (i.e., the edge distortion). In the initial state, only one site at the edge is excited. The lasing edge wave packet propagates without the backscattering at the edge disorder.
